# An Experiment in Sociology

**Published:** 1887-10-08

**Authors:** 


### The Hospital

A Weekly Institutional Journal of

**Science, Medicine, and Philanthropy.**

*For Hospitals, Asylums, Medical Practitioners, Students, Nurses, Families, Parishes, Congregations, and the Charitable Public.*

Vol. III. No. 54.] [October 8, 1887.

### An Experiment in Sociology.

Almost all kinds of animals have at some time or other proved their capacity for living together in society except medical students. They, as if driven asunder by some irresistible centrifugal force, have continued down to the present time to inhabit each his separate "cave," or "den," or "burrow," apparently neither knowing nor desiring anything different or better. The "den" must be admitted to have its own peculiar advantages and pleasures. To begin with, it may be the sole and exclusive possession of one particular "bear" into which no other "bruin" dare intrude without permission offered or requested. Or two kindred spirits may agree to a joint occupancy, and together or apart may smoke, or eat, or blow up the landlady according to the sovereign pleasure of each. When they please they may go out, and when they please they may come in. They may work early, or they may work late, or, if they are so inclined, they need not work at all. If the good woman of the house displeases their separate or conjoint high mightiness they can give her a week's notice and seek a retreat elsewhere. Should duns invade and be importunate, a cab after supper will remove the bears and their belongings to a sequestered haven where troublesome callers appear not and creditors are unknown. When remittances are liberal and good fellowship is in the ascendant, a dozen choice spirits can be assembled at a quiet hour, and wit and wisdom—sometimes accompanied by the rare music of the pianoforte, or the less rare but more inspiring strains of the clashing tongs and poker—reign triumphant, in spite of "bones" unlearnt, or of "text-books" neglected and despised. When, too, as sometimes happens even to medical students, the monthly cheque lingers on the way, and the watch has been taken to a place of great security; when the landlady ceases to be polite, and the maid-of-all-work brings in the boots altogether lacking in their accustomed polish; when the collar is worn a second day and then turned for a third; when the steak and potato, the jam-tart and the glass of Burton, have to give place to the sausage-roll with a slice of bread and the undiluted produce of the pump, then indeed—to shut to your door, draw out the pipe, whiff gently until cares are forgotten and "hope springs up once more eternal" in the student's breast—the "den" is felt to be a resting-place, a soothing spot, a refuge from every care.

So much behoved to be said in justice to a state of things which to many minds has attractions which are almost irresistible. There is about it an absolute freedom from restraint which to some men at the student period of life is dearer than any other pleasure they know. Let it not be imagined for a moment that all who love this Bohemian liberty are given to idleness, or indulgence in vulgar pleasures and dissipations. Nothing could be further from the facts of the case. The simple truth is, that those who want to be dissipated will be dissipated; whilst those who really love their work, and intend to do it, will not be tempted to riotous and wicked living whatever degree of freedom their circumstances may offer.

But there is another and less fascinating side to the question. Boys are not men; and whilst men of four or five and twenty may enjoy and be improved by freedom, mere youths of seventeen or eighteen are often ruined by it. Can anything be more foolish and dangerous than to send a boy, utterly inexperienced in the ways of life, when his passions are at the strongest, and his self-control is only beginning to be developed, into the very thick and hotbed of all that is most wicked, filthy, and relentless in modern civilisation? That many youths are sent into such a fiery furnace and come out unscathed is one of the marvels of human nature. That some are ruined and destroyed can surprise nobody—least of all those who have passed through the fire themselves.

All hospital teachers are well aware of the correctness of this description. Many of them have made such efforts as seemed possible in order to bring about a better state of things. It has been reserved for the *Middlesex* Hospital to make an experiment on a larger scale than has hitherto been tried, and to found a collegiate institution in connection with its medical school which shall offer to students some of the comforts of home with all the advantages of supervision, instruction, discipline, and order which a regular college affords. A large building has been opened in that part of the hospital grounds which borders on Cleveland Street, capable of accommodating thirty-three students. Here they will be boarded, lodged, and in all respects placed under the residential college system for ninety pounds a year. Each of them will have a separate room of large size for study, with bedroom curtained off. All will take dinner and other meals together in the college hall. There is a "common room" for social purposes and "house meetings"; and in almost every particular they will enjoy the social advantages characteristic of English university life. The building is a fine, well-planned structure, with good bath-room on each floor, and other arrangements for health, comfort, and convenience. The scheme is being worked by a Limited

Liability Company, with a subscribed capital of £6,000, the shares being £5 each. Most of the shareholders are either members of the Hospital Staff or in some way connected with the institution. The prime movers have been the Earl of Lathom, Chairman of Hospital Committee, and Major Alexander Ross, M.P., Deputy Chairman, and Chairman of the Council of The Hospitals Association. The hospital itself in no way shares in the risk of the undertaking. It is practically certain, however, that if the selected warden be equal to the duties of the post, not only will there be no loss to the promoters, but a handsome dividend in due time. But it is not with present or prospective dividends that we are concerned. As an effort to improve medical education, to preserve students from dangerous temptations, and to give to them that breadth, simplicity, ease, and polish which can only be acquired by constant contact with intellectual equals and superiors, the movement is deserving of unqualified approbation, and we heartily wish for it a prosperous and unclouded life.

Those who have had experience, as the present writer has, of both the residential and the non-residential systems, will unhesitatingly give the preference to the former in the case of very young or very inexperienced students, and also for those who for any reason have not had the advantages of early education and association with cultivated people. The regularity, discipline, and order of a collegiate institution have a restraining and disciplinary effect upon young men, which is very necessary at their time of life, and which perhaps cannot be obtained elsewhere. But in addition to that, and of quite as much importance, is that "licking into shape," as it may be called for want of a better phrase, which results from the daily contact on terms of equality with other young men of equal or superior culture and breeding. In cases where boys have had the advantages of a public school education this kind of influence may not be so necessary. But with many medical students no such training has been possible; and here it is that the residential system is invaluable. We do not for a moment say that all medical students should be compelled to enter the profession through the gates of a compulsory college residence. On the contrary, we would maintain the superior advantages of the non-residential system for certain classes of students. What we do affirm is, that for many youths residence means safety, education, and success, where non-residence would mean failure and ruin. Let the two systems flourish side by side, and let every medical school provide that parents and students shall always have the power of choice.

